# Risk-Factors for Exposure Associated With SARS-CoV-2 Detection After Recent Known or Potential COVID-19 Exposures Among Patients Seeking Medical Care at a Large Urban, Public Hospital in Fulton County, Georgia — A Cross-Sectional Investigation

**DOI:** 10.3389/fpubh.2022.809356

**Published:** 2022-03-24

**Authors:** Sarah E. Smith-Jeffcoat, Sadia Sleweon, Mitsuki Koh, George M. Khalil, Marcos C. Schechter, Paulina A. Rebolledo, Vyjayanti Kasinathan, Adam Hoffman, Rebecca Rossetti, Talya Shragai, Kevin O'Laughlin, Catherine C. Espinosa, Bettina Bankamp, Michael D. Bowen, Ashley Paulick, Amy S. Gargis, Jennifer M. Folster, Juliana da Silva, Caitlin Biedron, Rebekah J. Stewart, Yun F. Wang, Hannah L. Kirking, Jacqueline E. Tate, CDC COVID-19 Emergency Response GA-10 Field, Halie K. Miller

**Affiliations:** ^1^Centers for Disease Control and Prevention, Atlanta, GA, United States; ^2^Emory University School of Medicine, Division of Infectious Diseases, Atlanta, GA, United States; ^3^Grady Memorial Hospital, Atlanta, GA, United States; ^4^Emory University School of Public Health, Hubert Department of Global Health, Atlanta, GA, United States; ^5^Emory University Nell Hodgson Woodruff School of Nursing, Atlanta, GA, United States; ^6^Emory University School of Medicine, Pathology & Laboratory Medicine, Atlanta, GA, United States

**Keywords:** SARS-CoV-2, COVID-19, risk factors, exposure, underrepresented

## Abstract

We aimed to describe frequency of COVID-19 exposure risk factors among patients presenting for medical care at an urban, public hospital serving mostly uninsured/Medicare/Medicaid clients and risk factors associated with SARS-CoV-2 infection. Consenting, adult patients seeking care at a public hospital from August to November 2020 were enrolled in this cross-sectional investigation. Saliva, anterior nasal and nasopharyngeal swabs were collected and tested for SARS-CoV-2 using RT-PCR. Participant demographics, close contact, and activities ≤14 days prior to enrollment were collected through interview. Logistic regression was used to identify risk factors associated with testing positive for SARS-CoV-2. Among 1,078 participants, 51.8% were male, 57.0% were aged ≥50 years, 81.3% were non-Hispanic Black, and 7.6% had positive SARS-CoV-2 tests. Only 2.7% reported COVID-19 close contact ≤14 days before enrollment; this group had 6.79 adjusted odds of testing positive (95%CI = 2.78–16.62) than those without a reported exposure. Among participants who did not report COVID-19 close contact, working in proximity to ≥10 people (adjusted OR = 2.17; 95%CI = 1.03–4.55), choir practice (adjusted OR = 11.85; 95%CI = 1.44–97.91), traveling on a plane (adjusted OR = 5.78; 95%CI = 1.70–19.68), and not participating in an essential indoor activity (i.e., grocery shopping, public transit use, or visiting a healthcare facility; adjusted OR = 2.15; 95%CI = 1.07–4.30) were associated with increased odds of testing positive. Among this population of mostly Black, non-Hispanic participants seeking care at a public hospital, we found several activities associated with testing positive for SARS-CoV-2 infection in addition to close contact with a case. Understanding high-risk activities for SARS-CoV-2 infection among different communities is important for issuing awareness and prevention strategies.

## Introduction

Since January 2020, and as of January 2022, over 75,000,000 confirmed cases of COVID-19 and 800,000 deaths from COVID-19 have occurred in the United States (1). Furthermore, people of color have been disproportionally affected by SARS-CoV-2 infection and Black, non-Hispanic people have been underrepresented in COVID-19 investigations (2). SARS-CoV-2, the virus that causes COVID-19, reaches peak viral load soon after symptom onset which increases the likelihood of transmission and, consequentially, is the time period of focus for prevention strategies (3). Documented pre-symptomatic and asymptomatic transmission of SARS-CoV-2 reduces the effectiveness of preventing spread through isolation strategies (4). Initially, state and local governments enacted a myriad of restrictions on different types of indoor and outdoor activities to limit community transmission. Many of these decisions emerged from laboratory-based experiments or small outbreak investigations focused on COVID-19 transmission dynamics. More recently, several studies have investigated high-risk activities associated with testing positive for SARS-CoV-2; however, Black, non-Hispanic people represented only 2–13% of the participants (5–7). A few additional reports investigated exposures and potential high-risk activities among groups that have been disproportionately affected by the COVID-19 epidemic including people experiencing homelessness or incarceration (8, 9). More recently, the mass vaccination campaign in the U.S. has helped reduce COVID-19 severe illness and death (10). However, due to the introduction of the Omicron variant in the U.S., non-pharmaceutical interventions have again become essential to halting the pandemic.

As part of an investigation to understand the diagnostic performance of self-collected saliva and anterior nasal (AN) swabs compared to healthcare worker-collected nasopharyngeal (NP) swab, participants at a large, public hospital in Fulton County, Georgia, were interviewed about COVID-19 exposures, employment, and social activities that may be potential risk factors for exposure as part of a larger epidemiologic questionnaire at the time of specimen collection (11). In Fulton County, people identifying as Black, non-Hispanic, make up 44.5% of the total population and 31–53% of reported weekly COVID-19 cases are among Black, non-Hispanic people; however, nearly 16% of reported COVID-19 cases were missing race/ethnicity (12, 13). This study examined known exposures to persons with COVID-19, and potential unknown exposures (risk-factors) and estimated the risk for SARS-CoV-2 infection associated with such exposures among patients presenting for medical care at an urban, public hospital serving mostly low-income clients.

## Methods

### Recruitment and Enrollment

Enrollment, specimen collection, and testing procedures have been previously described (11). Briefly, this cross-sectional investigation enrolled patients aged ≥18 years presenting for medical care at the emergency department, preoperative screening clinic, or labor and delivery department at Grady Memorial Hospital during August–November 2020. Grady Memorial Hospital, an urban hospital in Fulton County, GA, serves the Atlanta metropolitan area including five surrounding counties and about two thirds of its clients are insured by Medicaid/Medicare or uninsured (14). Patients whose treating clinician ordered NP SARS-CoV-2 testing by reverse transcription polymerase chain reaction (RT-PCR)—for any reason (e.g., diagnostic or screening)—were eligible for study enrollment. Patients were excluded if they were unable to or declined consent, <18 years of age, unable to conduct self-collection of specimens, were enrolled previously during a prior visit, or if an NP swab was contraindicated. Trained interviewers as part of this investigation administered structured questionnaires in English or Spanish to collect patient demographic characteristics, known COVID-19 exposures, and risk factors for exposure to COVID-19 within the 14 days prior to enrollment. The sample size of this secondary analysis was based on the primary objective of the parent investigation (11).

Patients provided self-collected saliva and AN swab and a healthcare worker-collected NP swab. NP swab aliquots, saliva, and AN swabs were tested at the Centers for Disease Control and Prevention (CDC) using the CDC 2019-nCoV Real-Time RT-PCR Diagnostic Panel in accordance with the Emergency Use Authorization Instructions for Use (15).

### Data Management and Analysis

Data were entered and stored in the Research Electronic Data Capture (REDCap) software (Vanderbilt University, Nashville, TN) hosted at CDC. Our outcome of interest was any SARS-CoV-2 positive RT-PCR result from specimens collected on the same day as the interview and tested at CDC. A “known close contact” was defined as being within 6 ft of a known COVID-19 case for a total of ≥15 min over a 24-h period. “Close contact within the past 14 days” was defined as being a known close contact where the reported date of last exposure was ≤14 days before the date of the interview (16). “Essential worker” was defined as a participant who reported a job or job location that meets the U.S. Department of Homeland Security's definition of “essential critical infrastructure workforce” (17). “Essential activity” was defined in this analysis as grocery shopping, using public transit, or visiting a healthcare facility.

We described the demographics, close contacts, employment, and social activities reported by participants. Age was grouped into categories based on distribution and alignment with other reports. As we were interested in recent infections, participants reporting a prior positive COVID-19 test were excluded from the bivariate and multivariate analyses of potential risk factors and SARS-CoV-2 detection. Bivariate associations were assessed using Chi-square, Fishers exact, and Mann-Whitney *U* tests, where appropriate. An alpha of <0.05 was considered statistically significant. Logistic regression models were used to understand crude and adjusted odds ratios (cOR and aOR) of potential risk factors and testing positive for SARS-CoV-2. Age, gender, and race and Hispanic ethnicity were included in the multivariate models *a priori*. In the multivariate models' race/ethnicity variable, the non-Hispanic categories of American Indian/Alaska Native, Asian, Multiple Race, and Native Hawaiian/Pacific Islander were grouped with Unknown/Other/Refused due to small numbers. Other potential risk factors with a bivariate association *p* < 0.1 were considered for inclusion in the model. Collinearity of model covariates was assessed by reviewing correlation matrices, and the best fitting model was chosen based on Akaike's information criterion. Because there was some interaction of being a known close contact with the other risk factor variables and the outcome, we then repeated the analysis of potential risk factors and SARS-CoV-2 detection after removing participants who responded “yes” or “unknown” to having close contact with a COVID-19 case to better understand potential risk factors when the participant had no known close contact to a case. RStudio, R version 4.0.3 (Boston, MA) and SAS 9.4 (Cary, NC) were used for data analysis.

### Ethics

All participants provided written consent before enrollment. This activity was reviewed by CDC and was conducted consistent with applicable federal law and CDC policy (See e.g., 45 C.F.R. part 46, 21 C.F.R. part 56; 42 U.S.C. 241(d); 5 U.S.C. 552a; 44 U.S.C. 3501 et seq.). This investigation was determined to be an exempt public health activity by the Emory University Institutional Review Board and Grady Memorial Hospital Research Oversight Committee.

## Results

Among 1,078 enrolled participants, 559 (51.8%) were male, 516 (47.9%) were female, 2 (0.2%) were non-binary and 1 (0.1%) refused to answer (Table 1). Over half (57.0%) of participants were aged ≥50 years including 309 (28.7%) participants aged 50–59 years and 305 (28.3%) aged ≥60 years. Most participants identified as Black, non-Hispanic (*n* = 876; 81.3%) followed by White, non-Hispanic (*n* = 81; 7.5%) and Hispanic or Latino (*n* = 71; 6.6%).

**Table 1:** Frequency of exposures and potential risk factors for exposure to COVID-19 among persons accessing medical care and tested for SARS-CoV-2 by previous COVID-19 test result at a large urban, public hospital in Fulton County, GA, August-November 2020.

**Characteristic**	**No prior COVID-19 positive test**	**Prior positive COVID-19 test**	**Total**
	***n*** **(%)**	***n*** **(%)**	***n*** **(%)**
Overall	1,026 (95.0)	52 (5.0)	1,078 (100.0)
Gender			
Male	534 (95.5)	25 (4.5)	559 (51.8)
Female	489 (94.8)	27 (5.2)	516 (47.9)
Non-binary	2 (100.0)	0 (0.0)	2 (0.2)
Refused	1 (100.0)	0 (0.0)	1 (0.1)
Age group			
18–39	285 (94.4)	17 (5.6)	302 (28.0)
40–49	149 (92.0)	13 (8.0)	162 (15.0)
50–59	296 (95.8)	13 (4.2)	309 (28.7)
60+	296 (97.0)	9 (3.0)	305 (28.3)
Race/ethnicity			
American Indian/Alaska Native, non-Hispanic	2 (100.0)	0 (0.0)	2 (0.2)
Asian, non-Hispanic	9 (90.0)	1 (10.0)	10 (0.9)
Black, non-Hispanic	838 (95.7)	38 (4.3)	876 (81.3)
Hispanic/Latino	63 (88.7)	8 (11.3)	71 (6.6)
Multiple Race, non-Hispanic	15 (93.7)	1 (6.3)	16 (1.5)
Native Hawaiian/Pacific Islander, non-Hispanic	1 (100.0)	0 (0.0)	1 (0.1)
Unknown/other/refused, non-Hispanic	20 (95.8)	1 (4.8)	21 (1.9)
White, non-Hispanic	78 (96.3)	3 (3.7)	81 (7.5)
Exposure to COVID-19			
Ever known close contact to person who tested positive for COVID-19 (*n* = 1,075)			
Yes	111 (86.0)	18 (14.0)	129 (12.0)
No	864 (96.6)	30 (3.4)	894 (83.2)
Unknown	48 (92.3)	4 (7.7)	52 (4.8)
Days since known close contact (*n* = 129)			
≤ 14 days	28 (93.3)	2 (6.7)	30 (23.3)
>14 days	72 (85.1)	13 (14.9)	87 (65.9)
Unknown	11 (91.7)	1 (8.3)	12 (9.3)
In the past 14 days			
Housing Situation			
Single-family home	521 (95.4)	25 (4.6)	546 (50.6)
Multifamily home/shared housing with others	343 (93.7)	23 (6.3)	366 (34.0)
Move from house-to-house/shelter/no housing	127 (97.7)	3 (2.3)	130 (12.1)
Other or unknown	35 (97.2)	1 (2.8)	36 (3.3)
Employment			
Currently employed or worked in past 14 days			
Yes	349 (95.1)	18 (4.9)	367 (34.0)
No	677 (95.2)	34 (4.8)	711 (66.0)
Essential worker (n = 367)	349 (95.1)	18 (4.9)	367 (100)
Yes	284 (95.3)	14 (4.7)	298 (81.2)
No	65 (94.2)	4 (5.8)	69 (18.8)
Working location (n = 364)	347 (95.3)	17 (4.7)	364 (100)
Working from home 100%	26 (96.3)	1 (3.7)	27 (7.4)
Working outside of the home	295 (94.9)	16 (5.1)	311 (85.5)
Mix of working from home and outside of the home	26 (100.0)	0 (0.0)	26 (7.1)
If working outside the home, number of people working in close contact (n = 335)	319 (95.2)	16 (4.8)	335 (100.0)
<10	177 (96.7)	6 (3.3)	183 (54.6)
10–20	57 (93.4)	4 (6.6)	61 (18.2)
>20	85 (93.4)	6 (6.6)	91 (27.2)
Activities with potential risk for exposure to COVID-19			
Worship service	91 (96.8)	3 (3.2)	94 (8.7)
Funeral attendance	35 (97.2)	1 (2.8)	36 (3.3)
Choir practice	7 (99.4)	0 (0.0)	7 (0.6)
Rally or protest participation	5 (100.0)	0 (0.0)	5 (0.5)
Wedding attendance	6 (85.7)	1 (14.3)	7 (0.7)
Exercise class	24 (100.0)	0 (0.0)	24 (2.2)
Sports practice	12 (100.0)	0 (0.0)	12 (1.1)
Sporting event	11 (100.0)	0 (0.0)	11 (1.0)
Other event with >10 people	111 (91.0)	11 (9.0)	122 (11.4)
Indoor activities with potential risk for exposure to COVID-19			
Grocery shopping	712 (94.9)	38 (5.1)	750 (69.6)
Any shopping other than groceries	306 (94.4)	18 (5.6)	324 (30.1)
Eating/drinking at indoor restaurant	231 (92.4)	19 (7.6)	250 (23.2)
Used public transportation	339 (95.5)	16 (4.5)	355 (32.9)
Traveled on an airplane	21 (95.5)	1 (4.5)	22 (2.0)
Visited friends/family inside their home	324 (95.3)	16 (4.7)	340 (31.5)
Visited a healthcare facility (excluding current visit)	340 (95.2)	17 (4.8)	357 (33.1)

### Frequency of Exposure and Potential Risk Factors for Exposure to COVID-19

Most participants (*n* = 894; 83.2%) reported no known close contact to a COVID-19 case. Of the 129 (12%) participants who reported known close contact, 30 (23.3%) reported the last known exposure ≤ 14 days before enrollment (Table 1). Among those with known close contact, the close contact was a household member (*n* = 31; 24.3%), non-household family member (*n* = 26; 20.3%), or close acquaintance (i.e., friend, boyfriend, girlfriend, significant other) (*n* = 30; 23.4%) (Supplementary Table 1). More than half of the participants enrolled in the investigation reported living in a single-family home (*n* = 546; 50.6%), one third lived in a multifamily home or shared housing (*n* = 366; 34.0%), and 12.1% (*n* = 130) reported living in a shelter or unstable housing. Most participants reported always wearing a mask when leaving home to go inside another building (non-work location) (*n* = 864; 81.4%) and an additional 102 (9.6%) and 67 (6.3%) participants wore a mask most of the time and sometimes, respectively. A few participants reported never wearing a mask (*n* = 17; 1.6%).

One third of participants were employed or worked in the past 14 days (*n* = 367; 34.0%). The majority of employed participants reported working outside the home (*n* = 311; 85.5%) and working indoors (*n* = 209, 62.6%). Most common places of work for employed participants include working at a restaurant or bar (*n* = 63; 17.3%), health care facility (*n* = 45; 12.3%), or construction or landscaping service (*n* = 38; 10.4%). Among those employed, 81.2% (*n* = 298) reported jobs that met the definition of an essential worker. Most employed participants reported, on average, working in close contact each hour with <10 people (e.g., coworkers, clients; *n* = 183; 54.6%), 61 (18.2%) participants reported working in close contact with 10–20 people, and 91 (27.2%) participants reported working in close contact with >20 people. Most participants reported that they always wore a mask (*n* = 250; 75.1%), while 11.1% (*n* = 37) and 10.2% (*n* = 34) of participants reported wearing a mask most of the time and sometimes, respectively, while at work.

Nearly all (*n* = 982; 91.1%) reported participating in at least one of 16 queried social activities in the past 14 days (median = 2; range = 0–9 activities). Of the nine indoor or outdoor social activities queried, the most frequently reported activities were attending some other event with >10 people (11.4%; *n* = 122), attending a worship service (8.7%; *n* = 94), attending a funeral (3.4%; *n* = 36), and attending an exercise class (2.2%; *n* = 24) (Table 1). Of the seven indoor only activities, the most frequently reported activities were grocery shopping (69.6%; *n* = 750), visiting a healthcare facility (excluding current visit; 33.1%; *n* = 357), using public transportation (32.9%; *n* = 355), and visiting friends or family inside their home (31.5%; *n* = 340).

### Exposures and Risk Factors Associated With SARS-CoV-2 Infection

**Figure 1:**
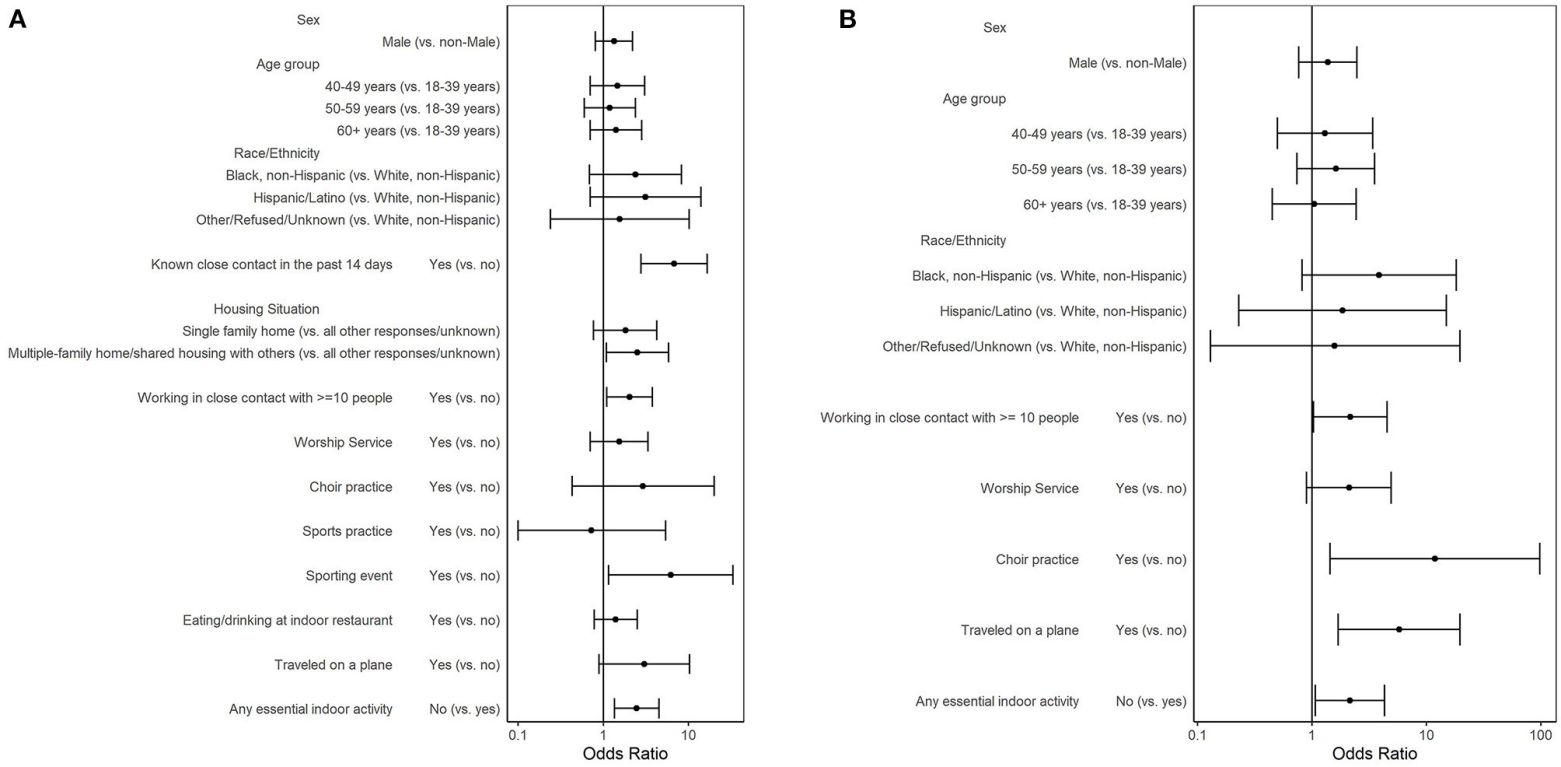
Adjusted odds ratios of a positive SARS-CoV-2 RT-PCR result: by COVID-19 exposure and by risk factors for COVID-19 exposure among **(A)** participants who did not report a prior positive COVID-19 test (*n* = 1,026) and **(B)** participants who did not report a prior positive SARS-CoV-2 test and had no known close contact to a COVID-19 case (*n* = 864); a study at a large urban, public hospital in Fulton County, GA, August-November 2020.

Fifty-two (4.8%) of 1,078 participants reported a previous positive COVID-19 test. Among 1,026 participants who did not report a previous positive test, 78 (7.6%) had a current positive SARS-CoV-2 RT-PCR result, 111 (10.8%) had close contact with a COVID-19 case, and 51 (5.0%) did not know if they had close contact with a COVID-19 case (Table 2). Responding “yes” or “unknown” to having close contact to COVID-19 case had 2.71 higher odds of a positive test compared to no close contact to COVID-19 case [crude Odds Ratio (cOR) = 2.71; 95%CI = 1.51–4.87 and cOR = 2.39; 95%CI = 1.03–5.55, respectively). Reporting known close contact with a COVID-19 case ≤14 days before enrollment had six times the odds of a positive test compared to those not reporting known close contact within 14 days (cOR = 6.38; 95%CI = 2.78–14.63). Participants working in close contact with ≥10 people had twice the odds of testing positive than those who worked with <10 people or who were not employed (cOR = 2.01; 95%CI = 1.15–3.52). Participants attending a sports practice or sporting event within 14 days had statistically higher odds of testing positive than those not attending (cOR = 4.17; 95%CI = 1.11–15.74 and 7.27; 95%CI = 2.08–25.39, respectively). In the selected multivariable model (AUC = 0.70; AIC= 539.90) controlling for age, gender, and race/ethnicity, known close contact with a COVID-19 case ≤ 14 days before enrollment (aOR = 6.79; 95%CI = 2.78–16.62), living in a multifamily home (aOR = 2.52; 95%CI = 1.09–5.86), working in close contact with ≥10 people (aOR = 2.04; 95%CI = 1.10–3.78), attending a sporting event (aOR = 6.25; 95%CI = 1.16–33.68), and not participating in any essential indoor activity (aOR = 2.47; 95%CI = 1.35–4.51) had statistically increased odds of testing positive for SARS-CoV-2 (Figure 1A).

**Table 2:** Associations between known and potential exposures to SARS-CoV-2 infected persons and testing positive for the virus; a study at a large urban, public hospital in Fulton County, GA, August-November 2020 (*n* = 1,026).

**Exposure or risk factor**	RT-PCR result^*^		***p*-Value^†^**	**cOR (95% CI)**	**aOR(95% CI)**
	**Positive *n* (%)**	**Negative *n* (%)**			
Overall	78 (7.6)	948 (92.4)			
Male gender					
Yes	44 (8.2)	490 (91.8)	0.4	1.21 (0.76, 1.93)	1.34 (0.81, 2.21)
No	34 (6.9)	458 (93.1)		ref	ref
Age group, years					
18–39	21 (7.4)	264 (92.6)	0.6	ref	ref
40–49	15 (10.1)	134 (89.9)		1.41 (0.70, 2.82)	1.47 (0.70, 3.07)
50–59	20 (6.8)	276 (93.2)		0.91 (0.48, 1.72)	1.19 (0.60, 2.38)
60+	22 (7.4)	274 (92.6)		1.01 (0.54, 1.88)	1.41 (0.70, 2.84)
Race/ethnicity					
Black, non-Hispanic	67 (8.0)	771 (92.0)	0.5	2.17 (0.67, 7.07)	2.40 (0.69, 8.30)
Hispanic/Latino	6 (9.5)	57 (90.5)		2.63 (0.63, 10.97)	3.14 (0.70, 14.06)
Other/refused/unknown	2 (4.3)	45 (95.7)		1.11 (0.18, 6.91)	1.56 (0.24, 10.29)
White, non-Hispanic	3 (3.8)	75 (96.2)		ref	ref
Ever had close contact to person who tested positive for COVID-19					
Yes	17 (15.3)	94 (84.7)	0.0008	2.71 (1.51, 4.87)	
No	54 (6.3)	810 (93.7)		ref	
Unknown	7 (13.7)	44 (86.3)		2.39 (1.03, 5.55)	
Known close contact in the past 14 days					
Yes	9 (32.1)	19 (67.9)	<0.0001	6.38 (2.78, 14.63)	6.79 (2.78, 16.62)
No	69 (6.9)	929 (93.1)		ref	ref
Known close contact relationship					
Household member	4 (17.4)	19 (82.6)	0.004	2.95 (0.97, 8.95)	
Family member, non-household	5 (22.7)	17 (77.3)		4.12 (1.47, 11.55)	
Other	8 (12.3)	57 (87.7)		1.97 (0.90, 4.31)	
No known close contact	61 (6.7)	855 (93.3)		ref	
In the past 14 days					
Housing situation					
Single family home	35 (6.7)	486 (93.3)	0.06	1.39 (0.63, 3.05)	1.82 (0.77, 4.26)
Multifamily home/shared housing with others	35 (10.2)	308 (89.8)		2.19 (0.99, 4.83)	2.52 (1.09, 5.86)
All other responses/unknown	8 (4.9)	154 (95.1)		ref	ref
Currently employed or worked in past 14 days					
Yes	34 (9.7)	315 (90.3)	0.06	1.56 (0.97, 2.48)	
No	44 (6.5)	633 (93.5)		ref	
Working outside of the home					
Working from home 100%	3 (11.5)	23 (88.5)	0.1	1.88 (0.54, 6.51)	
Mix of working from home and outside home	1 (3.9)	25 (96.1)		0.58 (0.08, 4.36)	
Working outside home	30 (10.2)	265 (89.8)		1.63 (1.01, 2.66)	
Not working	44 (6.5)	635 (93.54)		ref	
Working in close contact with ≥10 people					
Yes	18 (12.8)	123 (87.2)	0.01	2.01 (1.15, 3.52)	2.04 (1.10, 3.78)
No or not working	60 (6.8)	825 (93.2)		ref	ref
Activities with potential risk for exposure to COVID-19					
Worship service					
Yes	11 (12.1)	80 (87.9)	0.09	1.78 (0.90, 3.51)	1.54 (0.70, 3.37)
No	67 (7.2)	868 (92.8)		ref	ref
Choir practice					
Yes	2 (28.6)	5 (71.4)	0.09	4.96 (0.95, 26.01)	2.93 (0.43, 20.13)
No	76 (7.5)	943 (92.5)		ref	ref
Sports practice					
Yes	3 (25.0)	9 (75.0)	0.06	4.17 (1.11, 15.74)	0.72 (0.10, 5.40)
No	75 (7.4)	939 (92.6)		ref	ref
Sporting event					
Yes	4 (36.4)	7 (63.6)	0.007	7.27 (2.08, 25.39)	6.25 (1.16, 33.68)
No	74 (7.3)	941 (92.7)		ref	ref
Ate/drank at indoor restaurant					
Yes	24 (10.4)	207 (89.6)	0.07	1.59 (0.96, 2.64)	1.40 (0.78, 2.50)
No	54 (6.8)	741 (93.2)		ref	ref
Traveled on an airplane					
Yes	4 (19.1)	17 (80.9)	0.07	2.96 (0.97, 9.02)	3.04 (0.89, 10.34)
No	74 (7.4)	931 (92.6)		ref	ref
Any essential indoor activity^‡^					
Yes	61 (6.9)	823 (93.1)		ref	ref
No	17 (12.0)	125 (88.0)	0.03	1.83 (1.04, 3.24)	2.47 (1.35, 4.51)

* *RT-PCR positive was any positive RT-PCR for SARS-CoV-2 results from any of the submitted specimens (AN swab, saliva, NP swab) collected on the same day of the interview*.

† *p-values for Pearson chi-square test or fishers exact test, where appropriate*.

‡ *Any essential indoor activity was defined as reported grocery shopping, public transit use, or visit to a healthcare facility*.

*RT-PCR, real-time reverse transcriptase polymerase chain reaction; cOR, crude odds ratio; aOR, adjusted odds ratio*.

### Exposures and Risk Factors Associated With SARS-CoV-2 Infection Without Known Exposure

To understand risk factors when no known close contact was reported, we constructed an additional model where we excluded the 162 (15.8%) participants who responded “yes” or “unknown” to having close contact to a COVID-19 case. Among 864 participants who did not report a previous positive test nor a close contact to a COVID-19 case, 54 (6.3%) tested positive for SARS-CoV-2 (Table 3). Participants reporting attending worship service (cOR = 2.55; 95%CI = 1.19–5.46), choir practice (cOR = 15.54; 95%CI = 2.15–112.53), or traveling on an airplane (cOR = 4.55; 95%CI = 1.44–14.33) had statistically higher odds of testing positive for SARS-CoV-2 than those not reporting these activities. In the selected multivariable model (AUC = 0.67; AIC = 401.53) controlling for age, gender, and race/ethnicity, persons who worked in close contact with ≥10 people (aOR = 2.17; 95%CI = 1.03–4.55), attended choir practice (aOR = 11.85; 95%CI = 1.44–97.91), traveled on an airplane (aOR = 5.78; 95%CI = 1.70–19.68), or did not participate in an essential indoor activity (aOR = 2.15; 95%CI = 1.07–4.30) had statistically greater odds of testing positive for SARS-CoV-2 (Figure 1B).

**Table 3:** Association of risk factors for COVID-19 exposure and SARS-CoV-2 RT-PCR result among participants without a reported prior positive SARS-CoV-2 test and who reported no known close contact to a COVID-19 case; a study at a large urban, public hospital in Fulton County, GA, August-November 2020 (*n* = 864).

**Exposure or Risk Factor**	RT-PCR result^*^		**p-value^†^**	**cOR (95% CI)**	**aOR (95% CI)**
	**Positive n (%)**	**Negative n (%)**			
Overall	54 (6.3)	810 (93.7)			
Male gender					
Yes	32 (7.1)	422 (92.9)	0.3	1.34 (0.76, 2.34)	1.38 (0.77, 2.48)
No	22 (5.4)	388 (94.6)		ref	ref
Age group					
18–39	12 (5.3)	215 (94.7)	0.8	ref	ref
40–49	8 (6.7)	111 (93.3)		1.29 (0.51, 3.25)	1.30 (0.50, 3.39)
50–59	19 (7.5)	236 (92.5)		1.44 (0.68, 3.04)	1.62 (0.74, 3.54)
60+	15 (5.7)	248 (94.3)		1.08 (0.50, 2.37)	1.05 (0.45, 2.43)
Race/Ethnicity					
Black, non-Hispanic	49 (6.9)	661 (93.1)	0.6	2.30 (0.55, 9.68)	3.87 (0.82, 18.27)
Hispanic/Latino	2 (3.8)	51 (96.2)		1.24 (0.17, 9.12)	1.85 (0.23, 14.93)
Other/Refused/Unknown	1 (2.6)	37 (97.4)		0.84 (0.07, 9.56)	1.58 (0.13, 19.68)
White, non-Hispanic	2 (3.1)	62 (96.9)		ref	ref
In the past 14 days					
Working in close contact with ≥10 people					
Yes	11 (10.7)	92 (89.3)	0.05	2.00 (0.99, 4.01)	2.17 (1.03, 4.55)
No or not working	43 (5.7)	718 (94.3)		ref	ref
Activities with potential risk for exposure to COVID-19					
Worship Service					
Yes	9 (13.2)	59 (86.8)	0.03	2.55 (1.19, 5.46)	2.11 (0.90, 4.94)
No	45 (5.7)	751 (94.3)		ref	ref
Choir practice					
Yes	2 (50.0)	2 (50.0)	0.02	15.54 (2.15, 112.53)	11.85 (1.44, 97.91)
No	52 (6.1)	808 (93.9)		ref	ref
Indoor activities with potential risk for exposure to COVID-19					
Traveled on an airplane					
Yes	4 (22.2)	14 (77.8)	0.02	4.55 (1.44, 14.33)	5.78 (1.70, 19.68)
No	50 (5.9)	796 (94.1)		ref	ref
Any essential indoor activity^‡^					
Yes	42 (5.7)	703 (94.3)		ref	ref
No	12 (10.0)	108 (90.0)	0.07	1.86 (0.95, 3.64)	2.15 (1.07, 4.30)

* *RT-PCR positive was any positive RT-PCR for SARS-CoV-2 results from any of the submitted specimens (AN swab, saliva, NP swab) collected on the same day of the interview*.

† *p-values for Pearson chi-square test or fishers exact test, where appropriate*.

‡ *Any essential indoor activity was defined as reported grocery shopping, public transit use, or visit to a healthcare facility*.

*RT-PCR, real-time reverse transcriptase polymerase chain reaction; cOR, crude odds ratio; aOR, adjusted odds ratio*.

## Discussion

We aimed to describe frequency of COVID-19 exposure risk factors and risk factors associated with SARS-CoV-2 infection. This investigation, which focused on a low-income, urban population seeking medical care at a public hospital, found that most social activities were infrequent in the 14 days prior to enrollment. Grocery shopping, visiting a healthcare facility, and using public transit were the most frequently reported activities, all of which represent essential indoor activities and none of which were statistically associated with testing positive for SARS-CoV-2. Enrollment started just after the summer peak and ended right before Thanksgiving, during a period when testing percent positivity ranged from 3 to 5% in Fulton County (13). Participants' frequency of social activities may have been lower than normal as a reaction to the many cases and deaths in the surrounding community over the summer. Just over one third of participants reported being employed or working in the past 14 days, mostly as essential workers, highlighting vulnerability to SARS-CoV-2 among many of the participants. Like other investigations of exposure risk factors, we found that having close contact with a COVID-19 case especially in the 2 weeks prior to testing was a very strong risk factor for testing positive for SARS-CoV-2 (2, 7, 18–24). Living in a multifamily household and having close contact with non-household family members were associated with testing positive for COVID-19, further supporting the importance of these close social networks as a driver of SARS-CoV-2 transmission.

Contact tracing relies on patients identifying known individuals with whom they have had close contact and places they have visited. Additionally, it becomes much harder to name unknown individuals in the same room or within 6 feet of someone for ≥15 min. By excluding those who responded “yes” or “unknown” to having close contact to a COVID-19 case, we identified activities or work circumstances that could increase the risk for SARS-CoV-2 transmission to persons involved in such activities or circumstances. Among those who reported no known close contact to a COVID-19 case, working in close contact with ≥10 people, attending choir practice, traveling on an airplane, and not participating in an essential activity were associated with increased odds of testing positive for SARS-CoV-2. Being in close contact most days of the week with many different people, through one's job or living situation, increases the risk of SARS-CoV-2 transmission. While mask wearing was relatively high in this population, employers could increase efforts to promote similar prevention practices by encouraging time off to get vaccinated to reduce SARS-CoV-2 transmission in workplaces that require employees to work in close contact with each other or the public. In our analysis, during the 14 days prior to testing, choir practice showed a strong association with testing positive for SARS-CoV-2. In March 2020, an outbreak of COVID-19 among a church choir highlighted the ease of SARS-CoV-2 transmission among those singing indoors, in close range without masks (25). Singing is accompanied by jets of air from the lungs passing through moving vocal cords and out past the sinus, nasal, and oral cavities, all areas where SARS-CoV-2 has been detected. Singing has previously been shown to transmit other infectious diseases like TB (26). Encouraging virtual or outdoor, distanced, and masked choir sessions could help prevent SARS-CoV-2 transmission. Our finding that traveling by airplane was associated with increased odds of testing positive for SARS-CoV-2 could be from close contact on the airplane itself or from the many casual close contacts that one may experience in the act of traveling (e.g., waiting in line for ticket or luggage, traveling by taxi or metro to and from airport, etc.). A prospective cohort seroprevalence study found traveling by air was associated with incident seroconversion (6).

Previously published investigations have found some different social activities associated with a positive SARS-CoV-2 test. Several studies have found that dining at a restaurant or going to a bar or coffee shop were associated with testing positive for SARS-CoV-2 (6, 7). We did not find that indoor eating or drinking at a restaurant was associated with testing positive. However, participants in our investigation were recruited from a low-income, urban population seeking medical care at a public hospital which was substantially different from the populations included in the other previously published investigations that noted this association.

Our investigation had several limitations. First, participants were interviewed by individuals identifying themselves as CDC employees, which could have led to social desirability bias in the responses (the participants may have felt the need to provide a response they perceived to be satisfactory to CDC). Second, the cross-sectional design of this investigation does not allow us to make any claims of causation because we cannot say if the infection or the potential exposure from an activity occurred first. Third, this was a secondary analysis of a larger investigation; therefore, we were underpowered to detect small differences in social activities not commonly reported. Some of the associations that were detected were based on a very small number reporting the exposure and should be interpreted with caution. Fourth, interviewers did not explicitly ask about mask wearing during each individual activity, but rather an overall frequency of mask wearing when leaving the home to go inside another building. Lastly, our participants were recruited from a single public hospital in Fulton County, GA that serves largely low-income individuals, and may not represent the broader community in Atlanta or beyond.

This investigation among a population of mostly Black, non-Hispanic participants seeking care at a public hospital found several exposures and risk factors for exposure to COVID-19 associated with testing positive for SARS-CoV-2. Understanding the frequency of social activities and determining which activities increase the risk of SARS-CoV-2 transmission in different types of communities is important for both communicating with and preventing transmission among vulnerable populations. COVID-19 vaccine access and uptake has been slow in some populations at high risk of COVID-19 and there was high vaccine hesitancy among some of the most disproportionately affected populations in the United States (27, 28). Since Spring of 2021, there have been great strides in vaccination uptake within the United States which helps to prevent COVID-19 hospitalization and death (10). However, the partial immune evasion seen by the Omicron variant and waning immunity from initial vaccination series means that a return to focusing on non-pharmaceutical prevention measures will be key to reducing SARS-CoV-2 transmission in the Omicron variant era and beyond.

## Data Availability Statement

The datasets presented in this article are not readily available because data from this investigation were approved by CDC for COVID-19 pandemic response use and will be made public as aggregated data shared with CDC leadership and the public in the form of publications, presentations, or web content. Requests to access the datasets should be directed to SS-J, uyi7@cdc.gov.

## Ethics Statement

The studies involving human participants were reviewed and approved by Centers for Disease Control and Prevention, Emory University Institutional Review Board, and Grady Memorial Hospital Research Oversight Committee. The patients/participants provided their written consent to participate in this study.

## CDC COVID-19 Emergency Response GA-10 Field Team

Halie K. Miller, Ph.D.; AdeSubomi O. Adeyemo, PharmD; Anne C. Moorman, MPH; Brenda L. Bauman, MSPH; Kahaliah Joseph, MSc; Michelle O'Hegarty, Ph.D.; Nazia Kamal, Ph.D.; Mila Cohen, MSc; Amadea Britton, MD; Courtney T. Callahan, Ph.D.; Jamila Fonseka, MPH; Elfriede Agyemang, MD; Miriam J. Lawson, MS; Molly Deutsch-Feldman, Ph.D.; Tejpratap S. P. Tiwari, MD; Samira Sami, DrPH; Hong Tao, BS.

## Author Contributions

MS, PR, RS, YW, HK, and JT contributed to conceptualization, project design, and overall project leadership. SS-J, GK, VK, AH, RR, TS, KO'L, CE, BB, MB, AP, AG, JF, JS, CB, and the CDC COVID-19 Emergency Response GA-10 Field Team contributed to data specimen collection and specimen testing. SS-J, SS, MK, GK, and JT contributed to analysis and initial manuscript development. All authors contributed to manuscript editing and approval.

## Funding

Funding for this activity was provided by the U.S. Centers for Disease Control and Prevention and CDC Foundation Project #1085.

## Author Disclaimer

The findings and conclusions in this report are those of the authors and do not necessarily represent the official position of the U. S. Centers for Disease Control and Prevention (CDC).

## Conflict of Interest

YW, PR, VK, AH, and MS received funding for this study from the CDC Foundation. The remaining authors declare that the research was conducted in the absence of any commercial or financial relationships that could be construed as a potential conflict of interest.

## Publisher's Note

All claims expressed in this article are solely those of the authors and do not necessarily represent those of their affiliated organizations, or those of the publisher, the editors and the reviewers. Any product that may be evaluated in this article, or claim that may be made by its manufacturer, is not guaranteed or endorsed by the publisher.
