# Supplementary material for: Risk-Factors for Exposure Associated With SARS-CoV-2 Detection After Recent Known or Potential COVID-19 Exposures Among Patients Seeking Medical Care at a Large Urban, Public Hospital in Fulton County, Georgia — A Cross-Sectional Investigation
Source: Front Public Health. 2022 Mar 24;10:809356. doi: 10.3389/fpubh.2022.809356 (PMC9004481; doi:10.3389/fpubh.2022.809356)
Supplement: Supplementary file 1 [file Table_1.pdf]

Supplement Table 1: Full table of frequency of exposures and potential risk factors for exposure to COVID-19 among persons accessing medical care and tested for SARS-CoV-2 by previous COVID-19 test result at a large urban, public hospital in Fulton County, GA, August-November 2020

	**No Prior COVID-19 Positive Test** n (%)	**Prior Positive COVID-19 Test** n (%)	**Total** n (%)
**Exposure to COVID-19**			
Ever known close contact to person who tested positive for COVID-19 (n=1075)			
Yes	111 (86.0)	18 (14.0)	129 (12.0)
No	864 (96.6)	30 (3.4)	894 (83.2)
Unknown	48 (92.3)	4 (7.7)	52 (4.8)
Days since known close contact (n = 129)			
≤14 days	28 (93.3)	2 (6.7)	30 (23.3)
>14 days	72 (85.1)	13 (14.9)	87 (65.9)
Unknown	11 (91.7)	1 (8.3)	12 (9.3)
Known close contact relationship (n = 128)			
Household member	23 (74.2)	8 (25.8)	31 (24.3)
Family member, non-household	22 (84.6)	4 (15.4)	26 (20.3)
Friend, boyfriend/girlfriend, or significant other, non-household	27 (90.0)	3 (10.0)	30 (23.4)
Co-worker or person encountered at work	23 (95.8)	1 (4.2)	24 (18.8)
Person encountered in healthcare setting	3 (100.0)	0 (0.0)	3 (2.3)
Other	12 (85.7)	2 (14.3)	14 (10.9)
**In the past 14 days**			
Housing Situation			
Single-family home	521 (95.4)	25 (4.6)	546 (50.6)
Multifamily home/shared housing with others	343 (93.7)	23 (6.3)	366 (34.0)
Move from house-to-house/shelter/no housing	127 (97.7)	3 (2.3)	130 (12.1)
Other or unknown	35 (97.2)	1 (2.8)	36 (3.3)
Frequency of mask wearing when leaving home to go inside another building (nonwork location)			
Always	819 (94.8)	45 (5.2)	864 (80.1)
Most of the time	102 (100.0)	0 (0.0)	102 (9.5)
Sometimes	63 (94.0)	4 (6.0)	67 (6.2)
Never	16 (94.1)	1 (5.9)	17 (1.6)
Not Applicable	26 (92.9)	2 (7.1)	28 (2.6)
**Employment**			
Currently employed or worked in past 14 days			
Yes	349 (95.1)	18 (4.9)	367 (34.0)
No	677 (95.2)	34 (4.8)	711 (66.0)
Employment place (n = 365)			
Healthcare facility	43 (95.6)	2 (4.4)	45 (12.3)
Government/public service	15 (93.7)	1 (6.3)	16 (4.4)
Grocery store/gas station	13 (81.2)	3 (18.8)	16 (4.4)
Restaurant/bar	59 (93.7)	4 (6.3)	63 (17.3)
Retail	29 (96.7)	1 (3.3)	30 (8.2)
Cleaning service	16 (94.1)	1 (5.9)	17 (4.7)

Construction/landscaping service	35 (92.1)	3 (7.9)	38 (10.4)
Factory	19 (100.0)	0 (0.0)	19 (5.2)
Delivery services	13 (92.9)	1 (7.1)	14 (3.8)
School/daycare	13(100.0)	0 (0.0)	13 (3.6)
Other	94(100.0)	0 (0.0)	94 (25.7)
Essential worker (n = 367)	349 (95.1)	18 (4.9)	367 (100)
Yes	284 (95.3)	14 (4.7)	298 (81.2)
No	65 (94.2)	4 (5.8)	69 (18.8)
Working location (n = 364)	347 (95.3)	17 (4.7)	364 (100)
Working from home 100%	26 (96.3)	1 (3.7)	27 (7.4)
Working outside of the home	295 (94.9)	16 (5.1)	311 (85.5)
Mix of working from home and outside of the home	26 (100.0)	0 (0.0)	26 (7.1)
If working outside the home, work indoors or outdoors		15 (4.5)	
(n = 334)	319 (95.5)		334 (100)
Indoors	200 (62.7)	9 (17.3)	209 (62.6)
Outdoors	59 (96.7)	2 (3.3)	61 (18.3)
Mix	60 (93.7)	4 (6.3)	64 (19.1)
If working outside the home, number of people		16 (4.8)	
working in close contact (n = 335)	319 (95.2)		335 (100.0)
<10	177 (96.7)	6 (3.3)	183 (54.6)
10–20	57 (93.4)	4 (6.6)	61 (18.2)
>20	85 (93.4)	6 (6.6)	91 (27.2)
If working outside the home, frequency of mask		16 (4.8)	
wearing while at work (n=333)	317 (95.2)		333 (100.0)
Always	236 (74.4)	14 (26.9)	250 (75.1)
Most of the time	36 (97.3)	1 (2.7)	37 (11.1)
Sometimes	33 (97.1)	1 (2.9)	34 (10.2)
Never	12 (100.0)	0 (0.0)	12 (3.6)
**Activities with potential risk for exposure to COVID-19**			
Worship service	91 (96.8)	3 (3.2)	94 (8.7)
Outdoors	14 (93.3)	1 (6.7)	15 (1.4)
Indoors	77 (97.5)	2 (2.5)	79 (7.3)
Funeral attendance	35 (97.2)	1 (2.8)	36 (3.3)
Outdoors	17 (100.0)	0 (0.0)	17 (1.6)
Indoors	15 (100.0)	0 (0.0)	15 (1.4)
Indoors and outdoors	3 (99.7)	1 (0.3)	4 (0.4)
Choir practice	7 (99.4)	0 (0.0)	7 (0.6)
Outdoors	1 (100.0)	0 (0.0)	1 (0.1)
Indoors	6 (100.0)	0 (0.0)	6 (0.6)
Rally or protest participation	5 (100.0)	0 (0.0)	5 (0.5)
Outdoors	4 (100.0)	0 (0.0)	4 (0.4)
Indoors	1 (100.0)	0 (0.0)	1 (0.1)
Wedding attendance	6 (85.7)	1 (14.3)	7 (0.7)
Outdoors	1 (100.0)	0 (0.0)	1 (0.1)
Indoors	3 (75.0)	1 (25.0)	4 (0.4)
Indoors and outdoors	2 (100.0)	0 (0.0)	2 (0.2)
Exercise class	24 (100.0)	0 (0.0)	24 (2.2)
Outdoors	3 (100.0)	0 (0.0)	3 (0.3)
Indoors	20 (95.2)	1 (4.8)	21 (1.9)
Sports practice	12 (100.0)	0 (0.0)	12 (1.1)
Outdoors	4 (100.0)	0 (0.0)	4 (0.4)
Indoors	8 (100.0)	0 (0.0)	8 (0.7)
Sporting event	11 (100.0)	0 (0.0)	11 (1.0)

Outdoors	8 (100.0)	0 (0.0)	8 (0.7)
Indoors	3 (100.0)	0 (0.0)	3 (0.3)
Other event with >10 people	111 (91.0)	11 (9.0)	122 (11.4)
Outdoors	32 (100.0)	0 (0.0)	32 (3.0)
Indoors	62 (86.1)	10 (13.9)	72 (6.7)
Indoors and outdoors	17 (94.4)	1 (5.6)	18 (1.7)
Indoor activities with potential risk for exposure to COVID-19			
Grocery shopping	712 (94.9)	38 (5.1)	750 (69.6)
Any shopping other than groceries	306 (94.4)	18 (5.6)	324 (30.1)
Eating/drinking at indoor restaurant	231 (92.4)	19 (7.6)	250 (23.2)
Used public transportation	339 (95.5)	16 (4.5)	355 (32.9)
Travelled on an airplane	21 (95.5)	1 (4.5)	22 (2.0)
Visited friends/family inside their home	324 (95.3)	16 (4.7)	340 (31.5)
Visited a healthcare facility (excluding current visit)	340 (95.2)	17 (4.8)	357 (33.1)
